# Uterine Pains and Hemorrhage after Delivery

**Published:** 1855-10

**Authors:** W. Owen Brown

**Affiliations:** Caen, Corresponding member of the Medico-Chirurgical Society of Bruges; Providence, Rhode Island


*On Uterine Pain and Hemorrhage after Delivery.* Translated for the Boston Medical and Surgical Journal, from the French of Dr. Liegard, of Caen, Corresponding member of the Medico-Chirurgical Society of Bruges, by W. Owen Brown, M. D., of Providence, Rhode Island.

[The translator is indebted to Dr. S. Clapp, of Pawtuckett, Rhode Island, for the following interesting memoir, which Dr. C. met with during a late tour in Europe.]

The question proposed at the last meeting of the honorable and learned Medico-Chirurgical Society of Bruges, and so perfectly treated in the dissertation which has been judged worthy of the prize, related to the accidents which result from the implantation of the placenta upon the neck of the uterus (hemorrhage before delivery.) It seems natural and methodical to study now the accidents which present themselves after delivery—I mean hemorrhage and uterine pains.

The first has been the subject of numerous and important labors. Remedial means, various and more or less efficacious, have been pointed out and applied, among which compression of the aorta appears to hold the first rank, though this has as yet failed to fulfil all the indications. But that which science wanted, and which no practitioner has as yet sought to discover, was a preservative remedy, a means by which the causes of this formidable accident might with certainty be opposed, and consequently its occurrence prevented.

The second (uterine pains) has, on the contrary, been very much neglected by authors. The causes have been very vaguely indicated, and some soothing means only have been counselled, since these pains have been generally regarded as natural, inevitable, and even necessary, in order to effect the disgorgement of the contents of the uterus.

I propose, therefore, to-day to examine these two forms of accidents, and to indicate the means by the aid of which they are not to be combatted, but prevented *certainly* and *always*. This labor will be almost destitute of scientific theories; that which is important in practical medicine consisting much more in efficacious proceedings than in systems, more or less ingenious, but very often useless to the patient and physician.

1. Threatened hemorrhages after delivery.—An objection which presents itself at first to the mind of many, and which has been made to me by some accoucheurs of limited experience, is this: "How can it be foreseen that a woman will be attacked with hemorrhage after delivery? and if this accident cannot be forseen, why employ ourselves in preventing it? Is not this combating a veritable phantom? To this specious objection, witness what science and experience respond. When a woman has been the subject of grave and alarming hemorrhage, after her two or three first confinements, it is, perhaps, certain that this will be renewed in her approaching parturition; and moreover it happens almost always that this accident is much more formidable in the last confinement than the first. We conceive, in fact, that the ordinary cause of the loss of blood is the inertia, the want of contraction in the uterine walls becoming more and more decided in proportion as a series of gestations have debilitated and unfavorably relaxed the tissues of this organ.

Among the various means which science possesses and is able to oppose to this menacing danger, the most simple and the most efficacious is, undoubtedly, the ergot of rye, given some minutes before the birth of the child. But experience has demonstrated that the action of this medicine is not infallible, and that it is even completely inert in a certain number of subjects; so that it becomes very imprudent, in a case so grave, to rely wholly upon this means. For about fifteen years I have opposed this unfortunate predisposition effectually with the ergot of rye *before*, and cold injections into the umbilical vein *after* the accouchement; and this with such advantage, with a success so constant, that I now regard this hemorrhagic predisposition as a very simple affair, and one which does not inspire me with the least inquietude. This combined treatment appears so rational, that it is needless to demonstrate its efficacy by numerous observations. I will content myself by relating the two following cases, of which the first contains at once the proof of the usefulness of the two means united, and the insufficiency of the ergot of rye employed alone in the ordinary manner; the remarks relative to after pains come for the most part to rest upon the same foundation.

CASE 1.—Madame Douin, 28 years of age, of a sanguine nervous temperament and a feeble constitution, had safely arrived at the term for her fourth confinement. The former confinements, and particularly the last were followed immediately after the detachment of the placenta by a loss of blood so considerable that there appeared to be imminent danger of death. At the last confinement the hemorrhage was so great that, despite an enormous quantity of cold water poured forcibly upon the abdomen and thighs, it was not arrested until the occurrence of a very profound and prolonged syncope, and the patient was afterwards for a long time in a very feeble state, so that six weeks after, she was hardly able to raise her arms and turn herself in the bed, (she lived then in a town some leagues from Paris.) A point worthy of remark is, that her mother, who has borne three children, had been subject after each confinement to profuse hemorrhage. Numerous similar cases appear to demonstrate that this hemorrhagic predisposition is the result of a peculiar hereditary organic confirmation.

The parents, and particularly the husband, were much alarmed upon the occurrence of her fourth pregnancy. The latter came to me, stated the circumstances related above, and manifested extreme anxiety. The physician of the town, whom he had always previously employed, said that nothing could be done to remedy this unfortunate predisposition, and consequently he appeared greatly surprised when I gave him the positive assurance *that this time the hemorrhage should not take place!*

The pains commenced at 7 o'clock in the evening, (August 8, 1835,) and at 10 o'clock the labor was at the point of terminating. I then poured half a drachm of powdered ergot into a glass half full of sweetened water, and gave the whole between two pains. Ten minutes after, the child was expelled by the natural uterine pains. The uterus remained slightly contracted upon the placenta, but I saw nothing which indicated clearly the action of the medicine. Half an hour after the first, I administered a second half drachm dose of the ergot. I made light friction upon the lower part of the abdomeu, and soon occasional but feeble pains began to be felt, but the placenta still remained adherent. Gradually the pains lost all their energy, and at last ceased entirely. I feared that the womb becoming inert, the placenta would detach itself, and that the terrible hemorrhage of former confinements would be repeated. I prepared, therefore, promptly to make the injections. I poured a glass of vinegar into five glasses of very cold water, and injected about six fluid ounces (200 grammes) of this fluid into the umbilical vein. Immediately a cold sensation manifested itself in the fundus of the uterus, and almost at the same time a contraction was very evident in this organ. Some minutes after, I injected half a glass more of this acidulated water, and then the pains became more strong, the uterus contracted favorably, and I made with confidence moderate traction upon the cord. The placenta detached itself without the least difficulty, the uterine contraction continued without interruption, and there was no appearance of hemorrhage. This woman rose at the sixth day, and her health was afterwards very good.

Two years after, the same person was confined for the fifth time. I again had recourse to the ergot of rye, employing the same doses as before, about a quarter of an hour before the birth of the child, but after the uterine contractions appeared energetic. I determined this time to omit the injection. It was more than half an hour after the administration of the ergot, when I profited by a strong pain and made traction upon the cord. The placenta was easily detached, and came away perfectly intact; but almost as soon a profuse hemorrhage came on, and I promised myself, if ever this lady called on me again, under like circumstances, I should not fail to employ the injection.

Case 2.—A young woman of Mondeville, of a sanguine and lymphatic temperament, was pregnant for the third time, when she came, in the month of September, 1845, to inform me of her extreme anxiety. She told me that at her two first confinements, but especially at the second, she suffered such abundant hemorrhage, that despite a deluge of cold water which was poured upon her, the bleeding was not arrested until after a long and frightful syncope; and during many weeks she lay prostrated on her bed, unable to nourish her child, since the secretion of milk had failed entirely. The midwife had told her that if ever she became pregnant again, she would probably perish. I re-assured this woman against the pretended danger with such confidence that her courage was at once restored. She engaged to come, some days before the full term of her pregnancy, and reside at Caen, in order that we might give her the attention which her condition demanded.

On the 5th of the following January, which was the presumed period of her accouchement, she experienced some slight uterine pains, followed by a watery discharge from the vagina, which flowed little by little almost continually. As she was remaining in the country, I gave directions, which the midwife was charged to observe with great care. She was directed to give about gr. xxx. of ergot (2 grammes) a quarter of an hour before the birth of the child, and a like dose immediately after, she was not to make any traction upon the cord, but to leave that in order for me to make the injections. But during the following days, the uterine contractions disappeared almost entirely—being indicated only by some slight pains in the loins, accompanied and followed almost constantly with the flowing of limpid serosity, more or less considerable.

The 24th of January the mother of this woman came to inform me that for fifteen days past her daughter had been constantly in the same state; that until then she had been very feeble and fatigued, but that on the morning of the present day she had suffered many pains, one a little more marked, followed by a flowing of water more abundant than ordinary. I advised her to administer two or three grains (two decigrammes) of ergot, at intervals of ten or fifteen minutes, and to send for me if the labor made decided progress. They came for me at 8 o'clock in the evening; but instead of the uterine contractions having become energetic, it was impossible for me to appreciate, for an entire hour, with the hand placed over the fundus of the uterus, during the strongest pains felt by the woman, the slightest movement, the feeblest induration of the body of this organ. These throes consisted in a painful but very fugitive sensation in the lumbar region, accompanied always by the flowing, of which I have spoken, but which did not arrest at all the outcries of the patient. The head was yet above the superior strait, the os almost entirely dilated, but no appearances of the bag of waters. I administered thirty grains (2 grammes) of ergot. The pains in the loins became, perhaps, a little stronger, but there was evidently no active contraction. Three quarters of an hour after, the same amount of ergot repeated, produced no better effect than at first. At 11 o'clock the head was still in the same position; the neck was entirely obliterated and dilated. At 1 o'clock in the morning (January 25th,) the pains in the loins became stronger, and the waters flowed abundantly. The pains afterwards gradually diminished, and the woman slept. At 6 o'clock the pains revived a little, but at 7 they were almost imperceptible; the head had passed the superior strait, the vertex turned to the left acetabulum. It appeared as if a few slight contractions would suffice to expel the fœtus; but these continued to be absent, or almost so. The woman rose up, and, being supported, was made to walk a little. She then took three grammes of ergot, which proved equally unsuccessful. I sent to procure some more ergot, and also two grammes of pulverized savine. At 9 o'clock I had not felt any manifest contraction of the fundus of the uterus. I gave three grammes (about 45 grains) more of the ergot. Half an hour after, there had been no very apparent alteration, and I then administered 75 centigrammes (about 12 grains) of pulverized savine. A quarter of an hour after the last dose, I thought I could feel a slight uterine contraction; the woman experienced a stronger and more prolonged pain, and the head was soon in the passage. The chorion was prominent and firm. As the pains returned, though feeble and without well-pronounced uterine contraction, the woman was made to lie down. At 10 o'clock I ruptured the hard and resisting membrane, after many attempts to perforate them with the finger nails. The waters flowed in abundance. At a quarter past 10, the head being upon the point of passing the vulva, I gave about 30 grains of ergot, (2 grammes), and the fœtus was expelled a few moments after. The child was strong, but appeared to be suffering; it respired feebly, and its respiration was suspended for about a minute, when a long inspiration was made, and it again fell back into a state of frightful immobility. But having received the ordinary attentions, it cried, swallowed some sweetened water, and, in half an hour after, it appeared very well. The mother felt no pain in the lower part of the abdomen, and it was in vain that the hand sought to induce uterine contractions; everything announced, on the contrary, the imminent hazard of a dangerous flooding. Still, the placenta remained adherent, and the blood did not flow. At 11 o'clock I made the first injection into the umbilical vein. The cord was long, and the syringe contained only two or three fluid ounces (80 grammes). It was not until the third syringeful that the woman said that she felt an agreeable cold (this was her expression) in the fundus of the womb. I made a fourth injection, when the uterus directly became firm, and a slight traction brought away the placenta. During the following ten minutes I did not cease to make friction over the uterus with my hand, and to notice carefully the progress of its contraction. There was no hemorrhage. At 11½ o'clock, the woman complained of some uterine pains, and the womb never ceased to present to the hand which explored it, the sphere, which re-assures the accoucheur, and which is so justly denominated the globe. She nursed her child, and her health and strength were perfectly re-established.

Never have I seen a case of such profound inertia of the womb, or a labor with uterine contractions so futile! This woman, as she had carefully calculated the term of her pregnancy, ought to have been confined at the commencement of January. Was it the want of energy, or rather the complete nullity of uterine contractions, which occasioned this unusual prolongation of gestation and of labor? I am strongly induced to believe it was. But from whence came all the water which flowed almost without ceasing for sixteen days? Puzos, Lassus, and many other accoucheurs, have maintained that these liquid collections, which constitute a state of hydrometra, are situated between the chorion and the walls of the uterus. Baudelocque has denied the possibility of this. Nevertheless, it has not appeared to me probable that these waters came from the cavity of the amnois. The strength of the bag of waters, and their abundance, when, with difficulty, I was able to rupture the membranes, seemed to me to repel any such explanation.

I ought to observe, that in the two preceeding confinements this woman had had a similar flowing of water for nine or ten days preceding, and during all this time she had been exhausted by feeble pains, which seemed not at all to promote parturition.

From these two cases, and from many others, I believe it right to conclude that reason and experience prove, evidently, that injections of the umbilical vein, employed in concert with the ergot of rye, furnish an infallible means of preventing impending hemorrhage after confinement.

---

We believe there are numerous cases of dangerous, or even fatal flooding after delivery, where the placenta is expelled with the fœtus, or so soon after it as not to admit of injecting fluids into the uterus through the umbilical vein. Still, may not the suggestions in the text be of much practical utility, particularly in cases of adherent placenta?

In respect to the use of ergot of rye in cases of anticipated hemorrhage, Dr. Meigs says he gives it a few minutes previous to the termination of labor, "not for the purpose of aiding in the expulsion of the child or placenta, but by constringing the womb to save those dangerous losses." He says—"I scarcely ever omit such a precaution, for any patient of whom I am informed she floods after delivery."—(*Treat. on Obstetrics*, *p.* 347.)

